# An overview on small molecule-induced differentiation of mesenchymal stem cells into beta cells for diabetic therapy

**DOI:** 10.1186/s13287-019-1396-5

**Published:** 2019-09-23

**Authors:** Nimshitha Pavathuparambil Abdul Manaph, Kisha N. Sivanathan, Jodie Nitschke, Xin-Fu Zhou, Patrick T. Coates, Christopher John Drogemuller

**Affiliations:** 1 Central Northern Adelaide Renal and Transplantation Service, Royal Adelaide Hospital, Adelaide, South Australia 5000 Australia; 2 School of Pharmacy and Medical Sciences, Sansom Institute, University of South Australia, Adelaide, South Australia 5000 Australia; 3 School of Medicine, Faculty of Health Sciences, University of Adelaide, Adelaide, South Australia 5000 Australia; 4 Evergrande Center for Immunologic Diseases, Harvard Medical School and Brigham and Women’s Hospital, Boston, MA USA; 5 Neurological Disorders Research Center, Qatar Biomedical Research Institute, Hamad Bin Khalifa University, Qatar Foundation, Doha, Qatar

**Keywords:** Beta cells, Mesenchymal stem cells, Small molecules, Differentiation, Transplantation, Therapy

## Abstract

The field of regenerative medicine provides enormous opportunities for generating beta cells from different stem cell sources for cellular therapy. Even though insulin-secreting cells can be generated from a variety of stem cell types like pluripotent stem cells and embryonic stem cells, the ideal functional cells should be generated from patients’ own cells and expanded to considerable levels by non-integrative culture techniques. In terms of the ease of isolation, plasticity, and clinical translation to generate autologous cells, mesenchymal stem cell stands superior. Furthermore, small molecules offer a great advantage in terms of generating functional beta cells from stem cells. Research suggests that most of the mesenchymal stem cell-based protocols to generate pancreatic beta cells have small molecules in their cocktail. However, most of the protocols generate cells that mimic the characteristics of human beta cells, thereby generating “beta cell-like cells” as opposed to mature beta cells. Diabetic therapy becomes feasible only when there are robust, functional, and safe cells for replacing the damaged or lost beta cells. In this review, we discuss the current protocols used to generate beta cells from mesenchymal cells, with emphasis on small molecule-mediated conversion into insulin-producing beta cell-like cells. Our data and the data presented from the references within this review would suggest that although mesenchymal stem cells are an attractive cell type for cell therapy they are not readily converted into functional mature beta cells.

## Introduction

Beta cells are the major cells (70% of the total cells) in the islet of Langerhans of the pancreas, critical to store and release the hormone insulin to maintain glucose homeostasis [1]. Irregularities with the normal functioning of beta cells can lead to either type 1 diabetes mellitus (T1DM), where the islets are completely destroyed by the patient’s own immune system (autoimmune response), or type 2 diabetes mellitus (T2DM), where patients are unable to respond to insulin due to insulin resistance and inadequate production [2]. The American Diabetes Association reports that the confirmed cases of diabetes have exceeded 30.3 million in the USA, with a relatively larger population of 84 million classified as prediabetic [3]. Diabetes is one of the major chronic diseases which requires constant care throughout, as uncontrolled diabetes can affect the function of other organs and tissues leading to more severe conditions like diabetic ulcers, retinopathy, nephropathy, and neuropathy [4]. While islet transplantation has proven to be successful, the availability of donors, the quantity of functional cells recovered, requirement of immunosuppressive drugs, and cell loss after transplantation are limiting factors [5]. In order to overcome some of these obstacles, stem cell-derived beta cell therapy has been proposed as a feasible solution.

Current beta cell therapies are based on cells derived from embryonic stem cells (ESC), induced pluripotent stem cells (iPSC), or adult stem cells [6]. In order to generate ESC, a human embryo has to be destroyed in its early stages of development, thus involving ethical issues. iPSC require the cells to be reprogrammed to a pluripotent stage, followed by the germ layer-specific differentiation for the target cell [7]. In addition, pluripotent conversion can result in teratoma formation and most protocols incorporate transcription factors and integrating viral transgenes to induce pluripotency, which is not safe for clinical application [8]. Beta cell therapy is more feasible when patient-specific functional cells can be generated in larger quantities using clinically safe methods. Compared to ESC and iPSC, adult stem cell-like, mesenchymal stem cells (MSC) offer a good choice for generating differentiated cells for therapy.

MSC are immune privileged and highly plastic stem cells that can be isolated and cultured from the bone marrow, blood, skin, urine, fat, oral cavity, and even from the umbilical cord, placenta, and amniotic fluid [9]. In terms of the differentiation capability to generate germ layer cells, compared to other popular stem cells, MSC are similar to pluripotent stem cells [10]. Moreover, a single donor isolation can generate MSC in ample quantities (> 80 population doublings) for in vitro culture and differentiation [11]. The angiogenic and immunomodulatory properties of MSC are well reported, making them ideal candidates to generate functional beta cells for personalized medicine [12, 13]. Generating disease-specific cells from patients’ MSC using non-integrative methods not only avoids immunogenicity but also provides better engraftment and compatibility in vivo [14].

Non-integrative methods allow differentiation without incorporating foreign sequences into the host genome. Integrative methods like lentiviruses and retroviruses pose potential risk for tumor formation due to the genomic changes from transgenes [15]. Compared to integrative methods, a non-integrative method is safe and it poses less risk for tumor formation and genomic alteration [16]. For instance, adenoviral, sendai viral, episomal, mRNA, protein and chemical based deliveries are all safe and secure [17]. However, sendai viral and episomal methods have been reported mostly on iPSC-based differentiation and not on MSC [18]. Besides, they are laborious and methods utilizing adenoviruses do not work well on replicating cells like MSC as the viral genome is lost through subsequent passaging, resulting in low efficiency of differentiation. Protein-based methods are reliable but again, for mass production of cells, proteins and mRNA are required in large quantities making them laborious and expensive. However, small molecules or chemical-based differentiation is effective and safe as they can be introduced and manipulated effectively without genomic changes [19].

The safety and precision of small molecules in terms of their modification of signal pathways compared to the genomic alteration and integration by transcription factors has inspired researchers to think about the idea of incorporating small molecules to generate beta cells. Extensive investigations have been carried out to screen small molecules for generating beta cells as they can facilitate the generation of efficient functional cells for human therapy. A cocktail of nine small molecules and four recombinant proteins that specifically target the signal pathways related to pancreatic differentiation has been reported to generate functional beta cells from iPSC [20, 21]. Furthermore, most of the MSC-derived beta cell protocols also incorporate small molecules in their cocktail. In this review, we explore different small molecule-aided protocols reported on human MSC to generate insulin-secreting cells and the possibility of generating functional beta cells using small molecules alone. Focus is given on the small molecule-based protocols on human mesenchymal cells alone; however, the immunogenic properties of MSC are not discussed in this review.

## Plasticity of MSC

MSC are spindle-shaped cells identified to be originated from the perivascular linings of internal organs, demonstrating profound expandability and differentiation capability [22, 23]. As MSC are a subpopulation of cells that can be isolated from a variety of adult and perinatal tissues, the Mesenchymal and Tissue Stem Cell Committee of the International Society for Cellular Therapy guidelines has some strict criteria for their classification. The primary key genes and surface molecules that need to be expressed by these cells include CD73, CD90, and CD105, with minimal or no expression of CD11b, CD19, CD34, CD45, and HLA-DR [24]. In addition to surface molecule expression, the ability of MSC to differentiate into osteocytes, chondrocytes, and adipocytes is required for further confirmation [25]. Interestingly, according to the source of isolation, MSC express additional genes and surface molecules that increase their flexibility for differentiation.

MSC has been isolated from different adult sources like the peripheral blood, bone marrow, skin, foreskin, fat, heart, dental, skeletal muscle, lung, and pancreas. Irrespective of the different adult sources of MSC, beta cell differentiation has been only reported from few sources (Table 1). Furthermore, most in vivo research has been carried out using undifferentiated tissue-derived MSC. Even though the method of sample isolation is laborious, bone marrow-derived MSC are superior and well-studied in terms of generating differentiated cells for diabetic therapy [52]. Undifferentiated bone marrow-derived MSC have already shown promising preliminary results in clinical trials [53]. The anti-inflammatory and protective nature of the transplanted MSC improved the beta cell function and increased glycated hemoglobin in the subjects [53]. A similar trial has recently revealed that the transplanted mesenchymal cells not only differentiated into insulin-secreting cells but also did not incur immune reaction in the subjects, and therefore reveals promising future for MSC in diabetes therapy [54]. The transplanted autologous cells were able to integrate and differentiate into functional cells that demonstrated increased glucagon-stimulated C-peptide secretion, thereby decreasing the requirement of external insulin supply in 6 months [54]. Though pancreatic tissue-derived MSC is theoretically compliant to generate the robust cells, research suggests that the beta cell differentiation has not been efficient.

**Table 1:** Differential expression of markers on mesenchymal cells isolated from different sources

Type	Source of MSC	Positive expression	Negative expression	Beta cell differentiation reported	Reference
Adult tissues derived	Peripheral blood	CD105, CD90, CD73, CD73, CD44, CD90.1, CD29, CD105, CD106, CD140α	CD34, CD19, CD11b	No	[26]
	Bone marrow	CD105, CD13, CD140b, CD147, CD151, CD276, CD29, CD44, CD47, CD59, CD73, CD81, CD90, CD98	CD14, CD31, CD34, CD45	Yes	[27]
	Skin/foreskin	CD29, CD44, CD73, CD90, CD105, vimentin	CD34, CD45, HLADR	No	[28]
	Adipose	CD9, CD29, CD44, CD54, CD73, CD90, CD105, CD106, CD146, CD166	CD14, CD31, CD34, CD45, CD133, CD144, HLA-DR, STRO-1	Yes	[29]
	Urine	CD29, CD44, CD54, CD73, CD90, CD105, CD166, STRO-1, Oct-4, Klf-4, Sox-17, vimentin	CD41, HLA-DR	Yes	[30, 31]
	Heart	CD44, CD105, CD29, CD90	CD14, CD45, CD34, CD31	No	[32]
	Dental	CD13, CD29, CD44, CD49, CD73, CD90, CD146, STRO-1, Oct-3/4, NANOG, SSEA-3	CD14, CD31, CD34, CD45, HLADR	Yes	[33, 34]
	Skeletal muscle	CD29, CD44, CD49E, CD56, CD73, CD90, CD105, HLA-I	CD34, CD45	No	[35]
	Pancreas	CD105, CD90, CD73, CD44, CD29, CD13, nestin, vimentin, CD146, NG2, α-SMA, PDGF-R β	CD31, CD34, and CD45, CK19, CA19.9	Yes	[36]
	Lung	CD73, CD90, and CD105, vimentin, prolyl-4-hydroxylase	CD14, CD34, CD45	No	[37]
Pluripotent stem cell derived	ESC	CD29, CD44, CD73, CD105, SSEA-4,	CD34, CD45, HLADR	No	[38]
	iPSC	CD29, CD44, CD166, CD73, CD105, KDR, MSX2	CD34, CD45, HLADR	No	[39]
Birth related tissue derived	Wharton jelly	CD44, CD73, CD90, CD105, CD166	CD14, CD34, CD45	Yes	[40]
	Placenta	CD105, CD73, CD90c-kit, Thy-1, Oct-4, SOX2, hTERT, SSEA-1,3,4, TRA-1	CD34, CD45, CD14 or CD11b, CD19, HLA-DR	Yes	[41, 42]
	Umbilical cord	CD73, CD90, CD105, Oct-4, Nanog, ABCG2, Sox-2, Nestin	CD34, CD45, CD19, HLA-DR	Yes	[43, 44]
	Chorionic villi	CD44, CD117, CD105, α-SMA, CD49, CD146, CD106, CD166, Stro-1, vWF	CD34, CD45, CD19, HLA-DR	No	[45]
	Chorionic membrane	CD44, CD49, CD56, CD73, CD90, CD105	CD45, CD34, CD14, CD31, EPCAM, HLA-DR	No	[45]
	Cord blood	CD29, CD 73, CD105, CD44, Oct-4, Sox-1, Sox2, NANOG, ABCG2, Nestin	CD34, CD45	Yes	[44]
	Limb bud	CD13, CD29, CD44, CD90, CD105, CD106, SCA1, Runx2, SOX 9	CD3, CD5, CD11b, CD14, CD15, CD34, CD45, CD45RA, HLA-DR	No	[46, 47]
	Endometrium	CD73, CD90, CD105, CD166, HLA-ABC, Oct-4,	CD14, CD34, CD45, HLA-DR	No	[48]
	Amniotic membrane	CD73, CD90, CD105, Oct-4, SSEA-4, Tra-1	CD11b, CD14, CD19, CD79α, CD34, CD45, HLA-DR	No	[49, 50]
	Amniotic fluid	CD73, CD90, CD105, CD166, MHC class I, Oct-4, EA-1	CD 45, CD40, CD34, CD14, HLA-DR	Yes	[51]

After reprogramming gained popularity, embryonic stem cells and induced pluripotent stem cells also evolved as an interesting source for MSC. Furthermore, the MSC isolated from pluripotent stem cells have high expandability of > 120 population doublings without cellular senescence [55, 56]. However, the tumorigenicity and the embryonic nature of the pluripotent stem cell-derived MSC cannot be overlooked and beta cell differentiation has not been reported.

Mesenchymal cells from birth-related tissues have the great advantage in terms of the immunomodulation and plasticity for beta cell differentiation. Though allogenic, the immunosuppressive nature of the cells coupled with non-invasive method of isolation makes the perinatal-derived MSC an ideal candidate for diabetic therapy. Besides, the readiness in terms of the availability with minimal ethical issues makes them even more attractive. The main sources of MSC from perinatal tissues are the Wharton jelly, placenta, umbilical cord, chorionic villi, chorionic membrane, cord blood, limb bud, endometrium, amniotic fluid, and amniotic membrane [9]. MSC isolated from the umbilical cord and placenta have variable expression of pluripotent markers like Oct 3/4, SSEA1, and NANOG (differential gene expression given in Table 1), which undoubtedly contribute to their broad range of differentiation and proliferative capacity [57]. A human clinical trial involving the transplantation of amniotic fluid-derived MSC transplanted in diabetic subjects has shown protective effect on the damaged pancreatic cells of the patients, by interfering with insulin receptor/PI3K signaling pathway [58]. One-step differentiation of CD117+ cells of amniotic-derived MSC has shown the expression of PDX1 and other pancreatic-related genes [59]. Research also suggests that umbilical cord- and placenta-derived MSC have basal expression of pancreatic progenitors; however, this is again donor and protocol dependent [60]. Serum-free culture of chorionic villus-derived MSC has demonstrated the formation of islet-like clusters of GSIS quality [61]. Unlike the adult tissue-derived mesenchymal cells, most of the perinatal tissue-derived MSC have demonstrated robust trilineage differentiation exhibiting the plasticity similar to embryonic stem cells and pluripotent stem cells [62, 63].

Alternatively, functional glucose-responsive, insulin-secreting cells, derived from patients’ own MSC and generated in large numbers by small molecule-aided non-integrative methods, can be a better solution for curing diabetes [14]. Extensive literature is available for small molecule-based beta cell differentiation, especially on bone marrow-derived mesenchymal cells (Table 2).

**Table 2:** MSC-based protocols to generate beta cell-like cells

Source/type of MSC	Method	Endodermal differentiation	Pancreatic or endocrine differentiation	Maturation	Genes analyzed	Efficiency	In vivo testing reported	Functional analysis	Protocol duration in days	Reference
Umbilical cord	Extracellular matrix	–	–	–	NA	25.2	No	–	9	[64]
Umbilical cord	Small molecule	–	Nicotinamide in high glucose		C-peptide	–	No	–	7	[65]
Umbilical cord	ECM + small molecule + GF	–	High-glucose, retinoic acid, nicotinamide, EGF	FBS and exendin-4	Insulin, glucagon, Glut-2, PDX1, PAX4, NGN3	25	No	EM	15	[66]
Umbilical cord	Small molecule + peptide	–	High glucose, nicotinamide, exendin-4, 2-mercaptoethanol		Insulin,PDX1, glucagon	–	No	Nil	7	[67]
Umbilical cord blood	Plasmid electro transfer	–	PDX1 plasmid electro transfer		PDX1, Ngn3, NKX6.1	82.94 (PDX1 +ve cells)	No	Nil	10	[68]
Wharton jelly	Small molecule + GF	–	High glucose and RA, L-nicotinic acid and EGF	FBS and exendin-4	PDX1, NGN3, Glut2, and insulin	–	No	Dithizone staining, c-pep quantification	14	[69]
Placenta	Small molecule + peptide	–	l-Taurine and BSA	l-Taurine, nicotinamide, GLP-1, BSA	Insulin, glucagon, somatostatin	65	Yes	c-pep/insulin quantification, dithizone staining	20	[61]
Bone marrow	Small molecule + peptide	–	High glucose and nicotinamide	Exendin-4	PDX1, NGN3, NKX6.1, PAX4, Glut 2, glucagon, insulin	43	Yes	Dithizone staining, EM, c-pep quantification, calcium imaging	29	[70]
Bone marrow	Small molecule + GF	–	bFGF, high glucose, and nicotinamide	Nicotinamide, Activin A, and exendin 4	NKX6.1, ISL-1, NEUROD 1, Glut2, Pax6, PDX1, NGN3, insulin and glucagon	38.9	Yes	Dithizone staining, EM, c-pep quantification	15	[71]
Bone marrow	Adenoviral transfection		Transfection using PDX1 or VEGF		Insulin	50	Yes	–	2	[72]
Bone marrow	Small molecule + peptide	–	Nicotinamide and exendin-4		PDX1, NGN3, PAX4, IAPP, and insulin	20	Yes	–	5	[73]
Bone marrow	Small molecule + GF	–	BFGF, EGF	Nicotinamide, Activin, Betacellulin	PDX1, MAF-A,B, NGN3, PAX4, insulin, c-peptide	5	Yes	EM	18	[74]
Bone marrow	Viral transfection	–	Lentiviral transfection of miR-375 and anti-miR-9.		PDX1, NKX6., FoxA2, GCG, insulin, NGN3	85	No	Dithizone staining, c-pep quantification	21	[75]
Urine	Small molecules	IDE1, vitamin C	Indolactam V, retinoic acid	Nicotinamide, DAPT, SB203580	Sox-17, FoxA2, PDX1, NKX6.1, insulin, c-peptide	80	No	c-pep quantification	30	[76]
Dental	Small molecules + peptide	–	High glucose and retinoic acid	Low glucose, nicotinamide, EGF, Exendin A, Activin A, indolactam V	PDX1, NKx6.1, NGN3, Glut2, MAfA	–	No	c-pep quantification, dithizone staining	21	[77]
Adipose	Small molecule + peptide	Sodium butyrate and high glucose	l-Taurin	Nicotinamide and GLP-1	Hnf3β, TCF2, Sox17, PDX1, Ngn3, NeuroD, PAX4	–	No	–	16–18	[78]
Bone marrow	Retroviral infection	–	PDX1 infection and culturing in bFGF		PDX1, NeuroD1, NGN3, NKX6.1, ISl1	40–70	Yes	c-pep/insulin quantification	21	[72]
Bone marrow	Adenoviral transfection	–	PDX1 infection and culturing in GLP 1		NGN3, insulin, GK, Glut2, and glucagon	–	Yes	c-pep/insulin quantification, calcium imaging	14	[79]
Bone marrow	Serum-free culture	–	Serum-free media culture		NGN3, Brn4, NKX6.1, PAX6, and Isl1	33	Yes	c-pep quantification	6–7	[80]
	Serum free + small molecules + GF	–	1. High glucose and conophylline		PDX1, insulin, glucagon	~ 2.5 for all protocols	No	Dithizone staining, c-peptide quantification	15	[81]
		–	2. High glucose, trichostatin and GLP-1						10	
		–	3. High glucose and 2-ME, bFGF and EGF, betacellulin, nicotinamide, and activin						18	
Bone marrow	Adenoviral transfection	–	Infection of IPF1, HLXB9, and FOXA2		PDX1, NEUROD1, NKX6.2, Pax 6	50	No	–	14	[82]
Adipose	Small molecules + GF	–	High glucose, nicotinamide, Exendin, HGF, pentagastrin, activin A		Isl-1, Ipf-1, NGN, insulin, glucagon	10	Yes	–	3	[83]

Merged boxes represent the pancreatic and maturation stages induced together. Efficiency was calculated as the percentage of insulin- or c-peptide-positive cells at the end of the differentiation. *FBS* fetal bovine serum, *GF* growth factors, *bFGF* basic fibroblast growth factor, *EGF* epidermal growth factor, *VEGF* vascular endothelial growth factor, *HGF* hepatocyte growth factor

## Small molecules in regenerative medicine

In pharmacology and molecular biology, a small molecule is defined as “a compound of low molecular weight, which can diffuse into the cells to inhibit or improve a biological process” [84]. Cell therapy becomes feasible only when robust functional cells can be generated in unlimited quantities for transplantation. Stem cell therapy incorporates processes of reprogramming (process in which the somatic cell is converted back into pluripotent stage), transdifferentiation (direct conversion of one mature somatic cell into another), or dedifferentiation (conversion of mature somatic cell into their immature progenitor stage) to generate a desired cell type. Even though most of the protocols generate the required cells, many of them are non-functional and in some cases fail to restore the disease condition in vivo [85]. The demand for generating biologically active differentiated cells was a driving force for researchers to screen small molecules capable of directing cell-specific differentiation.

Initially, small molecules were used to modify the reprogramming efficiency of somatic cells by replacing transcription factors and further extensive studies have led to the discovery of molecules which can maintain the self-renewal and differentiation [86]. Not only can small molecules replace the transcription factors for reprogramming but can also be manipulated effectively to achieve robust differentiation [87]. Their effects have been specific and reversible [86]. Notable advantage of chemical formulations is that they are easy to handle and cost effective compared to transcription factor-mediated protocols [88]. Moreover, small molecules can aid the progression to feeder-free and serum-free protocols of stem cell culture [89]. Furthermore, they can reduce the use of non-compliant, animal-derived, and recombinant products [90]. The expression of stage-specific markers and the hierarchical targeting of signal pathways are important to generate mature beta cells from any type of stem cell [91]. Several key small molecules, which can potentially inhibit or activate the key beta cell signaling pathways, have already been reported [92].

## Small molecule induced MSC differentiation to beta cell-like cells

**Fig. 1:**
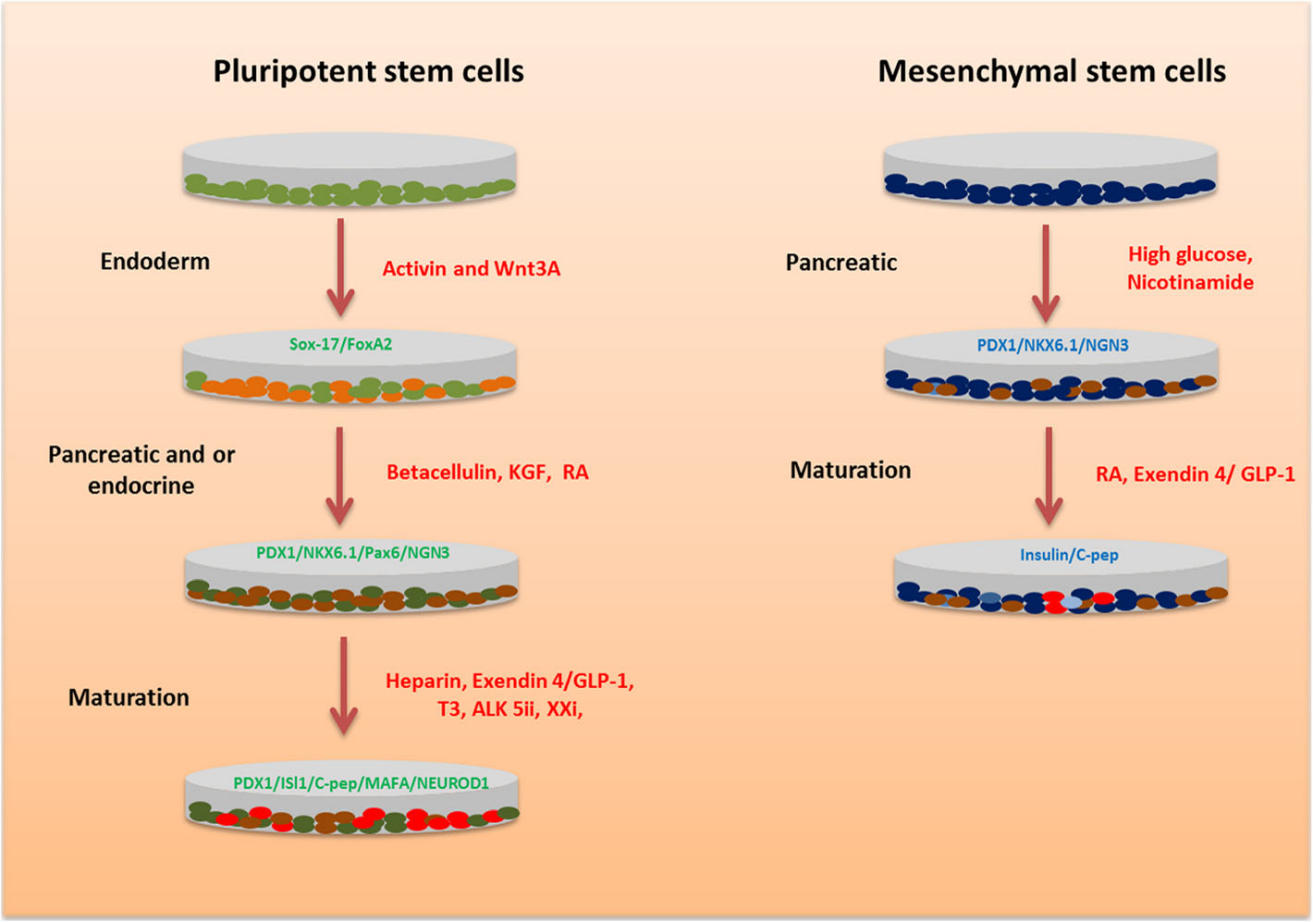
Stages of beta cell differentiation from pluripotent stem cells (PSC) and mesenchymal stem cells. Pluripotent stem cells like ESC/iPSC follow hierarchical targeting of signaling pathways to drive stage-specific genes to generate beta cells. MSC-based protocols do not follow an endoderm differentiation before the endocrine stage. KGF keratinocyte growth factor, T3 tri-iodo thyronine, XXi gamma secretase inhibitor, GLP-1 glucagon-like peptide 1

Beta cell differentiation from MSC follows two main steps. Firstly, the cells are differentiated into pancreatic progenitors followed by beta cell maturation (Fig. 1). Pancreatic progenitor differentiation was achieved mostly using nicotinamide with or without growth factors or peptides in high glucose culture (Table 2). In addition, chemicals like l-taurine and sodium butyrate also augmented the endocrine differentiation of MSC. The key markers analyzed during the pancreatic progenitor stage are PDX1, NKX6.1, and NGN3. The final maturation to beta-like cells was achieved by nicotinamide combined with exendin-4 or glucagon-like peptide-1 (GLP-1), and the critical genes analyzed included ISL1, insulin, and c-peptide. Compared to MSC differentiation, ESC- and iPSC-based protocols mainly comprise of three to five differentiation stages targeting specific signal pathways at each stage to achieve beta cell generation (Fig. 1). The different stages for pluripotent stem cells are the definitive endoderm (primitive tube and posterior foregut achieved separately or combined), pancreatic progenitor, and beta cell maturation. However, most of the MSC differentiation, unlike ESC- or iPSC-based differentiation, starts with a stage-specific pancreatic differentiation. Stage-specific endodermal differentiation is significant for generating efficient pancreatic lineage from MSC [93]. Mostly, the endoderm stage remains short that it may not be detected due to the strong signaling toward the next differentiation stage, which is pancreatic specification. Table 2 summarizes some of the important protocols for beta cell induction from MSC.

Conclusively, the MSC-based protocols take minimal time (7–25 days) to generate beta cell-like cells in vitro [94, 95]. Most of the protocols incorporate high glucose culture to drive the endocrine differentiation; however, the mechanism responsible remains unknown. Santos et al. have demonstrated that the differentiated cells have displayed abnormal phenotypes and consist of mixed population of endocrine cells [67]. Besides, some of the protocols have minimal expression of the key beta cell markers NKX6.1 and PDX1 [65, 73, 74, 81]. Irrespective of the large number of protocols, very few have succeeded to generate glucose-stimulated insulin secreted (GSIS) cells from MSC [81]. Many have failed to demonstrate glucose-specific insulin secretory responses, and have variable pancreatic marker expression (beta or alpha or beta and delta genes expressed together in the same cell) [96, 97].

Data suggest that mesenchymal stem cells are ideal protectants that aid in the survival of cell grafts after transplantation in vivo [98]. Islets, co-transplanted with MSC as protectants, were able to survive 2 months more than the animals transplanted with islets alone [99]. Results also suggest that the mesenchymal cells transplanted alone have also generated insulin-secreting cells and reversed the hyperglycemia in animal models by driving the repair of the damaged cells through the paracrine activity of MSC [100]. Follow-up of a clinical trial involving the infusion of MSC in T2DM patients has shown promising improvements in the blood glucose levels, thereby reducing the diabetic complications in the subjects [101].

## Discussion

**Fig. 2:**
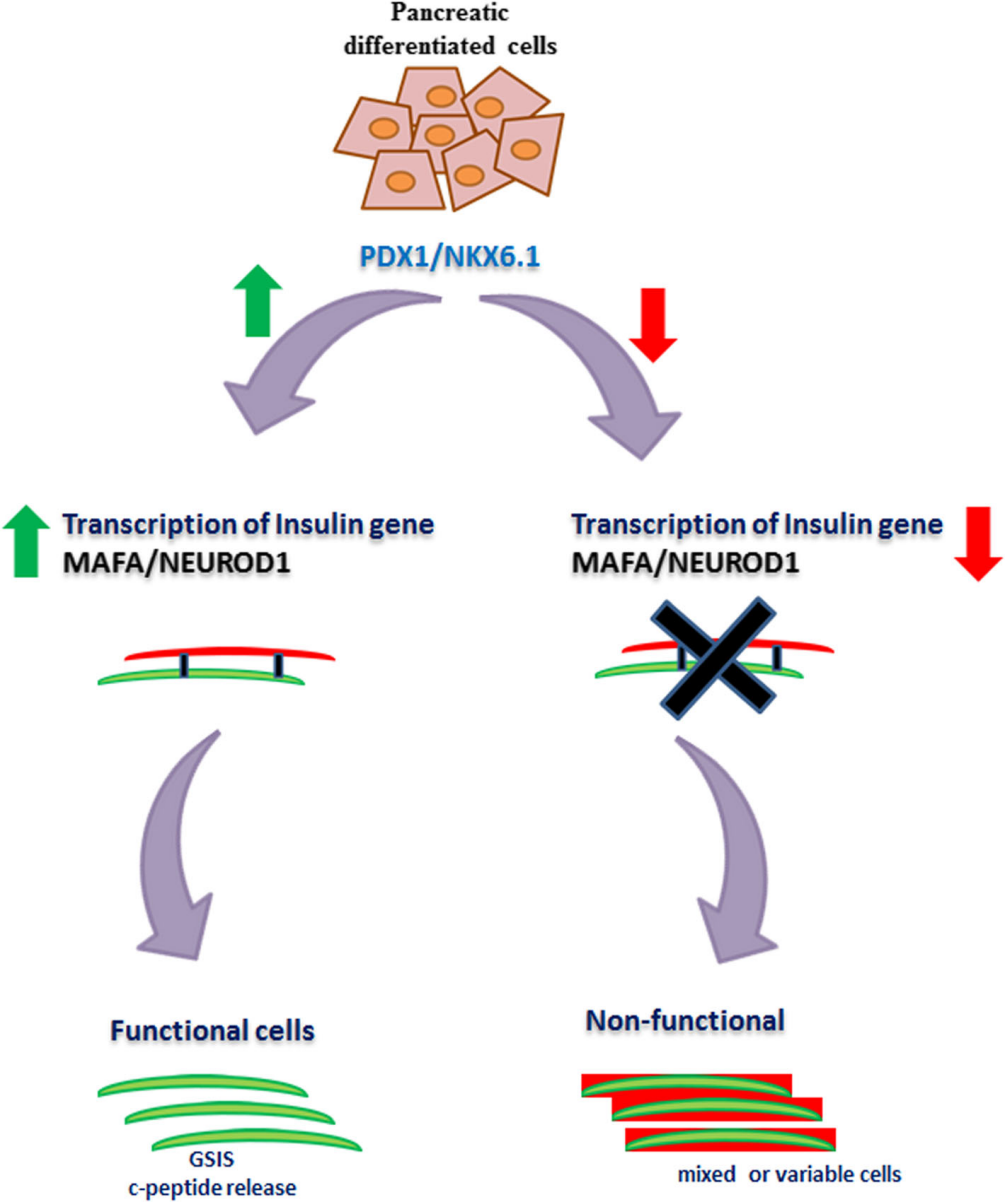
Synergistic action between PDX1 and NKX6.1 regulates C-peptide release. PDX1 functions together with NKX6.1 to initiate insulin transcription (or gene expression) during the differentiation, which results in the generation of functional beta cells. If PDX1 and NKX6.1 levels are high, the transcription of insulin gene will be increased leading to positive expression of MAFA and NEUROD1, thereby generating functional beta cells. On the other hand, reduced PDX1 and NKX6.1 gene levels will result in the decrease of the insulin gene further leading to the downstream generation of mixed or variable cells. The green arrow indicates increased levels of gene expression. The red arrow indicates decreased levels of gene expression. PDX1 duodenal homeobox 1, NKX6.1 NK6 homeobox 1, MAFA MAF BZIP transcription factor A, NEUROD1 neuronal differentiation 1

Current MSC-based protocols for beta cells generate a variable population of non-functional cells with abnormal phenotypes. Furthermore, the efficiency of beta cell differentiation is also lower, compared to the pluripotent stem cell-based differentiation (the average efficiency reported from the differentiation of iPSC and MSC is 80 and 60% respectively). iPSC-based research has helped to have a better understanding of the key genes necessary for the beta cell differentiation. For an efficient differentiation, the cells are converted into endoderm and further differentiated into pancreatic cells (which generate a mixture of endocrine cells) [102]. Recent chemical-based differentiation of patient-derived iPSC has revealed that the stage-specific expression of markers PDX1, NKX6.1, NEUROD1, and MAFA is critical for generating functional cells during the course of differentiation [20]. MSC-based protocols primarily focus on generating insulin-positive cells but do not focus on the stage-specific expression of genes. Furthermore, positive insulin gene expression may not necessarily drive the release of the hormone from the cells. In addition to insulin gene expression, the co-expression of PDX1 and NKX6.1 is equally important to generate mature beta cells [103]. Critical analysis suggests that irrespective of the PDX1 and NKX6.1 expression in some protocols, the cells are not functional. The percentages of cells expressing the key mature beta cell markers are also lower following the differentiation. One possible explanation for this phenomenon can be a low expression of PDX1/NKX6.1, regardless of the PDX1 expression. PDX1 functions together with NKX6.1 to initiate insulin transcription (or insulin gene expression) during the differentiation, which results in the release of the C-peptide molecule. If PDX1 and NKX6.1 levels are high, the transcription of insulin gene is augmented leading to elevated levels of C-peptide release (Fig. 2) [104]. On the other hand, reduced PDX1 and NKX6.1 gene levels result in the decrease of the insulin gene transcription leading to the generation of non-functional cells [105]. Therefore, the co-expression of markers PDX1 and NKX6.1 is critical to generate functional beta cells. In addition, mechanistic studies also reveal that the PDX1 drives the co-expression of NEUROD1 and MAFA, which in turn is also significant during the maturation of beta cells (Fig. 2) [91, 106]. Furthermore, to maintain the PDX1 levels, the synergistic expression of FoxA2 is also critical during the endodermal and pancreatic stages [107, 108]. The high efficiency of pluripotent stem cell-based differentiation is due to the FoxA2 expression in the early stages of beta cell differentiation. During the initial endoderm differentiation from pluripotent stem cells, Sox-17^+ve^/FoxA2^+ve^ cells are generated which in turn are converted to pancreatic cells. However, FoxA2 has not been analyzed in most MSC-based differentiation protocols. Interestingly, a single study incorporating lentiviral transfection of micoRNAs has shown the FoxA2 expression during the differentiation, thereby increasing the efficiency of conversion to 85% [75].

Nicotinamide has been a critical component for the generation of pancreatic cells from MSC (Table 2). A single study involving nicotinamide alone has generated insulin-secreting cells from MSC in high glucose culture [65]. Cells transplanted together with nicotinamide have generated insulin-positive cells in vivo aiding in the homing of cells in the pancreas [109]. Results suggest that nicotinamide aids in the generation of insulin-positive cells from undifferentiated cells; however, the exact mechanism of nicotinamide-based conversion has been unknown [110]. Some small molecules can function effectively in the presence of or combined with other factors. Synergistic action between nicotinamide and bone morphogenetic protein-4 augments the levels of PDX1, whereas their effects are not considerable when incorporated alone [111]. Furthermore, activin A combined with betacellulin helps to maintain PDX1 expression during pancreatic differentiation and maturation in ESC [112]. Interestingly, nicotinamide with betacellulin has also improved the differentiation in bone marrow-derived MSC [74].

**Fig. 3:**
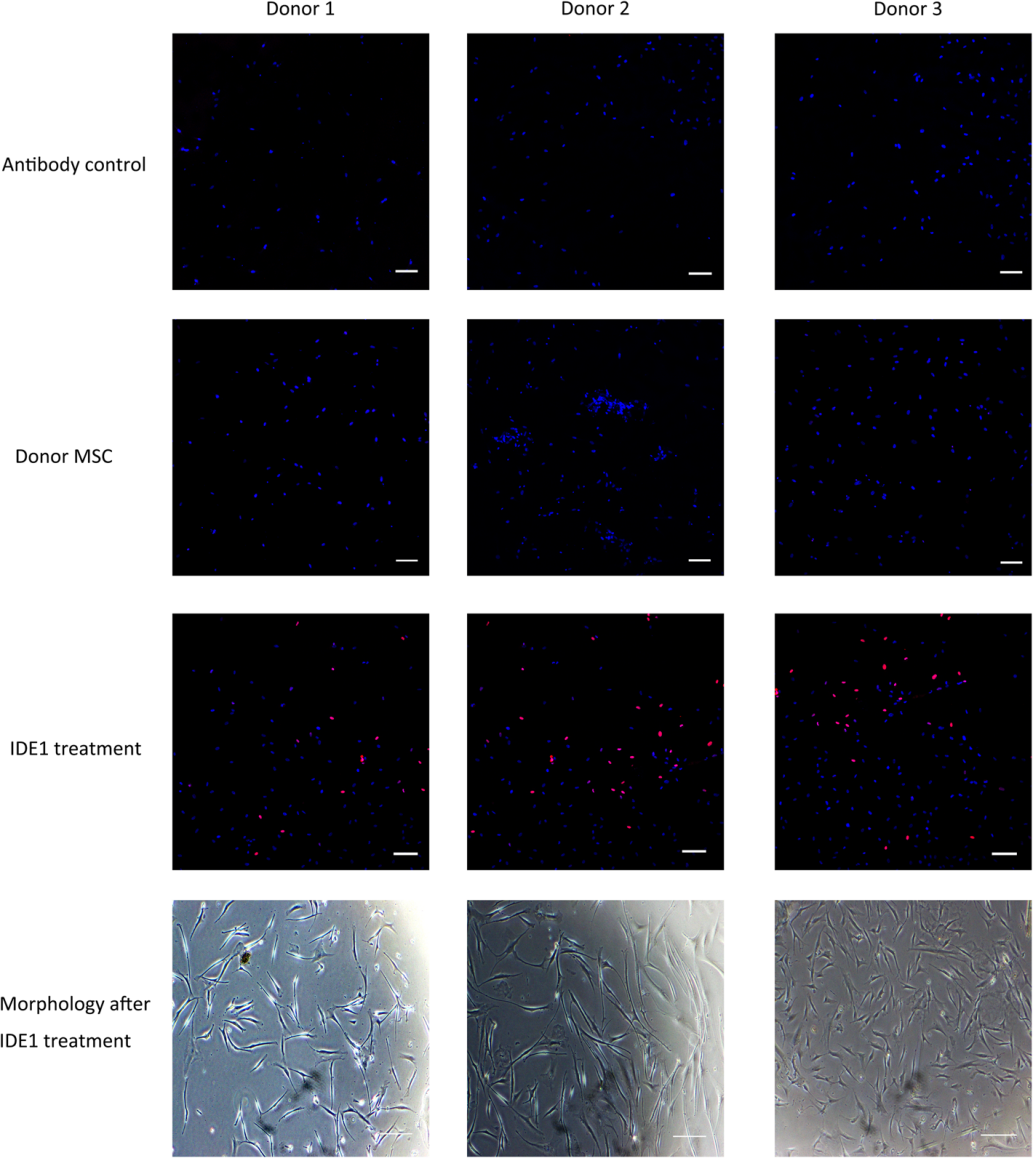
Sox-17 expression on bone marrow-derived mesenchymal stem cells after the treatment with 100 μM IDE1. Immunostaining of cells demonstrates the variable expression of the marker on different MSC donor cells. Confocal images taken by Olympus FV3000 fluorescent microscope at × 10 magnification, scale bar 100 μm. The blue color indicates individual nuclear staining of the cells by DAPI, and the red color indicates the Sox-17 expression. Antibody control indicates the sample with secondary antibody alone (no Sox-17 antibody added)

The variable low percentage of pancreatic cell differentiation from MSC suggests an impure donor culture and therefore generating a homogenous culture before differentiation is critical. Epigenetic modifiers like 5-aza cytidine and Rg108, which belong to the well-known class of epigenetic modifiers, can facilitate the generation of a homogenous culture [113]. Bone marrow MSC treated with 5-aza cytidine have been shown to generate GSIS-positive cells in a high glucose culture [114]. Moreover, mesodermal in origin, MSC could be less plastic to direct pancreatic differentiation, as beta cells are endoderm-derived. This germ layer hindrance can also account for the low efficiency of current MSC-based differentiation as most protocols follow the pancreatic differentiation first. One possible solution to achieve successful differentiation from MSC is to bring the cells initially into the same nature that of beta cells (in this context, endoderm). Moreover, this germ layer interconversion can be easily achieved by small molecules [115]. Furthermore, a stage-specific endodermal conversion and late stream differentiation using sequential treatment with small molecules was reported to generate 85% of functional beta cells from urine-derived MSC [76]. These results suggest that the endodermal differentiation is critical to generate efficient beta cells from MSC. Recently, a potent molecule, IDE1 (inducer of definitive endoderm 1), has been reported to generate a homogenous endodermal population (Sox-17^+ve^/FoxA2^+ve^) in vitro [116]. IDE1 can replace activin A suggesting that proper screening can identify molecules that can replace recombinant proteins, and this, in turn, can generate a clinically compliant protocol for therapy [117]. Besides, our in-lab analysis of bone marrow-derived MSC has revealed a positive expression of Sox-17 with the treatment of IDE1 (Fig. 3).

**Fig. 4:**
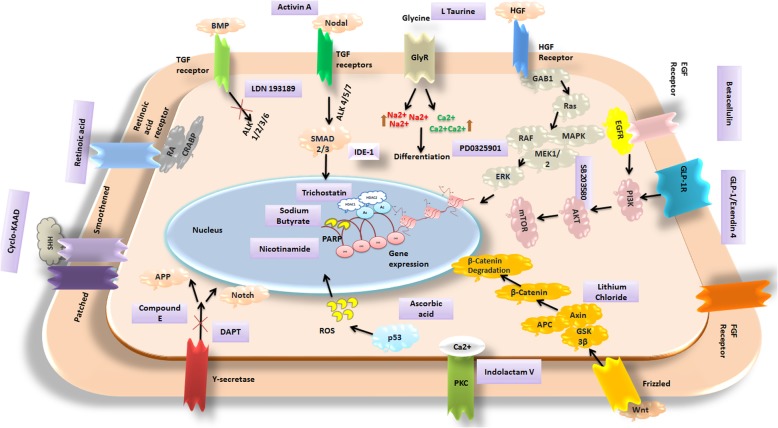
Important signaling pathways that govern the differentiation of pancreatic conversion and the mechanism of action of different small molecules and peptides reported on pancreatic differentiation from MSC. Violet boxes represent the small molecule that governs the representative signaling pathways or epigenetic modification. The substrates or proteins in the pathway are represented in a rounded shape. HGF hepatocyte growth factor, SHH sonic hedgehog, APP amyloid precursor protein, RA retinoic acid, ROS reactive oxygen species, EGF epidermal growth factor, HDAC1 histone deacetylase inhibitor 1, HDAC2 histone deacetylase inhibitor 2, Ac acetylation marks, FGF fibroblast growth factor, PKC protein kinase C, BMP bone morphogenic protein, TGF β transforming growth factor, GlyR glycine receptor, GLP-1 glucagon-like protein 1

Simultaneous induction and inhibition of pathways with epigenetic modification are also crucial for accomplishing robust differentiation. Comparing the different protocols of the beta cell differentiation from different sources, the key signaling pathways that are crucial in the differentiation of MSC are BMP, Wnt, Nodal, Notch, Shh, retinoic acid, EGF, and FGF (Fig. 4). Favoring Wnt and Nodal signaling has been reported to generate pancreatic cells from bone marrow- and fat-derived MSC. Selective inhibition of ALK pathway (ALK 1, 2, 3, 6) coupled with Notch inhibition is ideal for the generation of robust endodermal cells from fat-derived MSC [93]. However, the differentiation of pancreatic endocrine cells from iPSC is reported to be dose dependent on Notch inhibition. As such, the effects are comparable in MSC, which needs to be further investigated. Increasing the EGF signaling by betacellulin has favorable effects on the generation of PDX1+ cells from MSC [74]. The proteins and substrates linked to the EGF signaling (PI3K, AKT, mTOR) and HGF signaling (MEK, MAPK, and ERK) play important role in the proliferation, differentiation, and cell survival of pancreatic lineage. A small molecule SB0203580 that interferes with both EGF and HGF pathways has replaced the need for the recombinant supply in culture during the differentiation of urine-derived MSC [76]. In addition, notch and gamma secretase inhibition also appears to be crucial for hindering the transcription of genes non-ideal for beta cell differentiation. Furthermore, epigenetic changes also influence the differentiation considerably. However, limited information is available on the epigenetic implications during pancreatic differentiation in MSC. Small molecules like trichostatin and sodium butyrate help to maintain the necessary acetylation patterns of genes favoring the differentiation and repressing the ones for hepatic fate [118]. Histone methyl transferase inhibitors promote the FoxA2-dependent pancreatic fate and downstream transcription of INS, MAFA genes for endocrine specification [119].

The various methods used for confirming the purity of beta cells from MSC are not well advanced. Apart from the typical stage-specific analysis of genes at the DNA and RNA level, not much improvement has been made to characterize them in vitro like ESC- or iPSC-derived cells. Selective gene analysis (PDX1, NGN3, C-peptide, and insulin) and occasional dithizone staining are performed to characterize the differentiated cells from MSC [120]. Furthermore, microscopic analysis (transmission electron microscopy TEM) can be performed to check the insulin-secreting granular structure [120, 121]. Insulin and C-peptide release with different glucose responses can be used to confirm the functional purity of induced cells for therapeutics. Recently, calcium channel analysis has become popular for assessing the in vitro functionality of stem cell-derived beta cells [121]. Nevertheless, in vivo model studies have not been performed for most protocols (Table 2), and where they were conducted, long-term analyses need to be carried out.

Irrespective of the different sources of MSC, most of the beta cell differentiation was reported from the bone marrow and umbilical cord (Tables 1 and 2). Bone marrow-derived MSC are universal and stand superior in terms of generating clinically graded cells for human therapy [122]. Furthermore, perinatal tissue-derived cells secrete more c-peptide than bone marrow-derived cells after differentiation [123]. This can be due to naïve nature of the cells, which accounts for the flexibility for differentiation. However, translation of perinatal tissue-derived mesenchymal cells needs further extensive studies related to immunogenicity.

## Conclusion

Apart from the recent advances, diabetic cell therapy has been facing challenges for the successful transplantation of grafts [124]. One possible treatment for diabetes is the transplantation of glucose-responsive insulin-secreting cells into the body [124]. Small molecule-aided, non-integrative methods can be utilized to produce large numbers of functional, glucose-responsive, insulin-secreting beta cells derived from patients’ own cells [14]. However, beta cell therapy requires ample quantities of functional cells for transplantation (at least 10^9^ for a single graft) [125]. The population of target cells that are required for beta cell therapies is proportional to the amount of parent cells isolated. Even though the bone marrow-derived MSC are the pioneer cells in the clinical research, the method of sample isolation is invasive [31]. Further research is required to isolate and characterize MSC so they can be isolated from less invasive samples like the skin, blood, and urine. In particular, MSC isolated from urine are an attractive donor-derived source for regenerative therapy [31]. Research also suggests the generation of functional beta cells from urine-derived MSC by small molecule treatment alone [76]. Improvements in differentiation can be achieved by screening and thereby incorporating small molecules to drive stand-alone and co-expression of functional markers in other tissue-derived MSC. Analysis needs to be done to screen and test small molecules that can favor the differentiation of functional beta cells from MSC.

Current protocols mostly generate cells that exhibit some biological characteristics of beta cells. The cells express some of the key beta cell markers; however, further optimization is required to generate mature, good-quality cells for therapy. MSC differentiation combined with the appropriate screening of small molecules can possibly generate any desired cell type for autologous personalized therapy. Moreover, clinically graded MSC are commercially available enabling screening and parallel differentiation to establish feasibility of the different protocols for clinical translation. Manufacturing beta cells from MSC using small molecules can facilitate the development of clinically compliant cells and superior grade cells for therapy [126]. As chemical-based differentiation can aid in the expression of the key functional genes during differentiation, the cells will be able to perform functionally equivalent to mature beta cells after transplantation [127]. Nevertheless, immature pancreatic cells from mesenchymal cells (FoxA2+/PDX1+/NKX6.1+) transplanted in diabetic mouse were able to integrate and differentiate into mature cells (INS+) and rapidly reverse the disease condition [128]. Likewise, differentiated beta cell-like cells from the donor MSC can integrate, survive, and efficiently function after the clinical transplantation in patients. As transdifferentiated or differentiated using small molecules from patients’ own cells, there will be no rejection after the transplantation of MSC-derived beta cells. Besides, compared to pluripotent stem cell-differentiated cells, the chances of tumorigenicity will be less. Furthermore, the toxicity or residual effect from the small molecule treatment will be minimal as the concentrations administered for differentiation is small (details of small molecules used for differentiation is provided in Table 3). The normal concentration of small molecules tested for beta cell differentiation ranges from 10 nM–10 mM, which is comparatively lesser than the recommended small molecule drug concentrations for diabetic treatment by US Food and Administration (FDA) in human body [129]. However, once the proper differentiation will be achieved by the treatment of the small molecules, the differentiated cells will be maintained in normal beta cell culture conditions (RPMI with serum and antibiotics). Furthermore, this will help to remove any residual effects from the chemicals prior to transplantation. MSC can be easily maintained under feeder-free and serum-free conditions compared to other widely used stem cells [130]. Although non-differentiated donor-derived MSC do not impart an immune reaction after transplantation, studies need to be conducted to determine if the small molecule treatment will cause immunogenicity.

**Table 3:** Small molecules and peptides used for beta cell differentiation from MSC. Physiological or biochemical actions and substitutes for the small molecules/peptides used to generate beta cells from different tissue-derived MSC

Small molecule or peptide—name	Structure	Mode of action and effect	Possible substitute	Ref
Nicotinamide		PARP inhibition, drives beta cell outgrowth	Not known	[131]
IDE-1 (inducer of definitive endoderm 1)		Favors nodal signaling, induces Sox-17-reporter	IDE-2	[86, 116, 132]
l-Taurine (2-aminoethanesulfonic acid)		Cryoprotective agent binding with glycine receptors, involved in calcium signaling	Not known	[133]
Vitamin C		Reducing p53 levels and lightening senescence, thereby inducing complete reprogramming	Not known	[134]
ILV (indolactam V)		Protein kinase C activation, can induce pancreatic progenitor differentiation	PbdU	[135, 136]
RA (retinoic acid)		Receptor for retinoic acid receptors; promotes differentiation into neurons, glia cells, adipocytes	Not known	[136, 137]
SB203580		MAP kinase (MAPKAP kinase-2 and P38 MAPK) inhibitor, heat shock protein inhibition	PD98059, U0216	[138]
Trichostatin		Class 1 and II histone deacetylase inhibitor, removes acetyl groups from the histone tails.	Not known	[139]
Sodium butyrate		Class I inhibitor of histone deacetylases	Not known	[140]
Lithium chloride		Promotes Wnt signaling, blocks glycogen synthase kinase 3-β pathway	SB216763, CHIR99021	[141]
PD0325901		MEK/ERK kinase pathway inhibitor	Not known	[142]
LDN 193189		Inhibitor of ALK 1,2,3,6 pathways	Not known	[143]
DAPT		Gamma secretase inhibition	Compound -E	[121]
Pentagastrin		CCKB agonist, expands beta cell mass.	Not known	[144]
GLP-1		Augments maturation, belongs to the group of hormones that reduce blood glucose by binding with GLP receptors	Exendin, Exenatide, semaglutide	[145, 146]
Exendin-4		GLP receptors agonist	GLP-1	[147]

## Data Availability

All data generated and/or analyzed during this study are included in this published article.

## Abbreviations

ESC: Embryonic stem cells
FoxA2: Forkhead box A2
IDE-1: Inducer of definitive endoderm 1
iPSC: Induced pluripotent stem cells
ISL1: ISL LIM homeobox 1
MAFA: MAF BZIP transcription factor A
MSC: Mesenchymal stem cells
NEUROD1: Neuronal differentiation 1
NGN3: Neurogenin-3
NKX6.1: NK6 homeobox 1
Oct ¾: Octamer-binding transcription factor 3/4
PDX1: Pancreatic and duodenal homeobox 1
Sox-17: SRY-Box 17
SSEA-1: Stage-specific embryonic antigen 1

## References

[1] Fu Z, Gilbert ER, Liu D (2013). Regulation of insulin synthesis and secretion and pancreatic beta-cell dysfunction in diabetes. Current Diabetes Rev.

[2] Best M, Carroll M, Hanley NA, Piper HK (2008). Embryonic stem cells to beta-cells by understanding pancreas development. Mol Cell Endocrinol.

[3] Economic costs of diabetes in the U.S. in 2017. Diabetes Care 2018, 41(5):917–928. 10.2337/dci18-0007 PMC5911784 29567642

[4] Kharroubi AT, Darwish HM (2015). Diabetes mellitus: the epidemic of the century. World J Diabetes.

[5] Shahjalal HM, Abdal Dayem A, Lim KM, Jeon T-I, Cho S-G (2018). Generation of pancreatic β cells for treatment of diabetes: advances and challenges. Stem Cell Res Ther.

[6] Eguizabal C, Montserrat N, Veiga A, Izpisua Belmonte JC (2013). Dedifferentiation, transdifferentiation, and reprogramming: future directions in regenerative medicine. Semin Reprod Med.

[7] Patel M, Yang S (2010). Advances in reprogramming somatic cells to induced pluripotent stem cells. Stem Cell Rev.

[8] Lee AS, Tang C, Hong WX, Park S, Bazalova-Carter M, Nelson G, Sanchez-Freire V, Bakerman I, Zhang W, Neofytou E (2017). Brief report: external beam radiation therapy for the treatment of human pluripotent stem cell-derived teratomas. Stem Cells.

[9] Hass R, Kasper C, Böhm S, Jacobs R (2011). Different populations and sources of human mesenchymal stem cells (MSC): a comparison of adult and neonatal tissue-derived MSC. Cell Commun Signal.

[10] Grove JE, Bruscia E, Krause DS (2004). Plasticity of bone marrow-derived stem cells. Stem Cells.

[11] Trounson A (2004). Stem cells, plasticity and cancer - uncomfortable bed fellows. Development.

[12] Watt SM, Gullo F, van der Garde M, Markeson D, Camicia R, Khoo CP, Zwaginga JJ (2013). The angiogenic properties of mesenchymal stem/stromal cells and their therapeutic potential. British Med Bull.

[13] Atoui R, Chiu RCJ (2012). Concise review: immunomodulatory properties of mesenchymal stem cells in cellular transplantation: update, controversies, and unknowns. Stem Cells Transl Med.

[14] Daley GQ (2007). Towards the generation of patient-specific pluripotent stem cells for combined gene and cell therapy of hematologic disorders. Hematology Am Soc Hematol Educ Program.

[15] Rao MS, Malik N (2012). Assessing iPSC reprogramming methods for their suitability in translational medicine. J Cell Biochem.

[16] Schlaeger TM, Daheron L, Brickler TR, Entwisle S, Chan K, Cianci A, DeVine A, Ettenger A, Fitzgerald K, Godfrey M (2015). A comparison of non-integrating reprogramming methods. Nat Biotechnol.

[17] Zhou YY, Zeng F (2013). Integration-free methods for generating induced pluripotent stem cells. Genomics Proteomics Bioinformatics.

[18] Nakanishi M, Otsu M (2012). Development of Sendai virus vectors and their potential applications in gene therapy and regenerative medicine. Curr Gene Ther.

[19] Han Y-C, Lim Y, Duffieldl MD, Li H, Liu J, Abdul Manaph NP, Yang M, Keating DJ, Zhou X-F (2016). Direct reprogramming of mouse fibroblasts to neural stem cells by small molecules. Stem Cells Int.

[20] Millman JR, Xie C, Van Dervort A, Gurtler M, Pagliuca FW, Melton DA (2016). Generation of stem cell-derived beta-cells from patients with type 1 diabetes. Nat Commun.

[21] Pagliuca FW, Millman JR, Gurtler M, Segel M, Van Dervort A, Ryu JH, Peterson QP, Greiner D, Melton DA (2014). Generation of functional human pancreatic beta cells in vitro. Cell.

[22] Mendes LF, Pirraco RP, Szymczyk W, Frias AM, Santos TC, Reis RL, Marques AP (2012). Perivascular-like cells contribute to the stability of the vascular network of osteogenic tissue formed from cell sheet-based constructs. PLoS One.

[23] Al Madhoun A, Alkandari S, Ali H, Carrio N, Atari M, Bitar MS, Al-Mulla F (2018). Chemically defined conditions mediate an efficient induction of mesodermal lineage from human umbilical cord- and bone marrow- mesenchymal stem cells and dental pulp pluripotent-like stem cells. Cell Reprogram.

[24] Dominici M, Le Blanc K, Mueller I, Slaper-Cortenbach I, Marini F, Krause D, Deans R, Keating A, Prockop D, Horwitz E (2006). Minimal criteria for defining multipotent mesenchymal stromal cells. The International Society for Cellular Therapy position statement. Cytotherapy.

[25] Baghaei K, Hashemi SM, Tokhanbigli S, Asadi Rad A, Assadzadeh-Aghdaei H, Sharifian A, Zali MR (2017). Isolation, differentiation, and characterization of mesenchymal stem cells from human bone marrow. Gastroenterol Hepatol Bed Bench.

[26] Li S, Huang K-J, Wu J-C, Hu MS, Sanyal M, Hu M, Longaker MT, Lorenz HP (2015). Peripheral blood-derived mesenchymal stem cells: candidate cells responsible for healing critical-sized calvarial bone defects. Stem Cells Transl Med.

[27] Amati E, Perbellini O, Rotta G, Bernardi M, Chieregato K, Sella S, Rodeghiero F, Ruggeri M, Astori G (2018). High-throughput immunophenotypic characterization of bone marrow- and cord blood-derived mesenchymal stromal cells reveals common and differentially expressed markers: identification of angiotensin-converting enzyme (CD143) as a marker differentially expressed between adult and perinatal tissue sources. Stem Cell Res Ther.

[28] Park JR, Kim E, Yang J, Lee H, Hong SH, Woo HM, Park SM, Na S, Yang SR (2015). Isolation of human dermis derived mesenchymal stem cells using explants culture method: expansion and phenotypical characterization. Cell Tissue Bank.

[29] Schäffler A, Büchler C (2007). Concise review: adipose tissue-derived stromal cells—basic and clinical implications for novel cell-based therapies. Stem Cells.

[30] Zhang D, Wei G, Li P, Zhou X, Zhang Y (2014). Urine-derived stem cells: a novel and versatile progenitor source for cell-based therapy and regenerative medicine. Genes Dis.

[31] Pavathuparambil Abdul Manaph N, Al-Hawwas M, Bobrovskaya L, Coates PT, Zhou X-F (2018). Urine-derived cells for human cell therapy. Stem Cell Res Ther.

[32] Pilato CA, Stadiotti I, Maione AS, Saverio V, Catto V, Tundo F, Dello Russo A, Tondo C, Pompilio G, Casella M (2018). Isolation and characterization of cardiac mesenchymal stromal cells from endomyocardial bioptic samples of arrhythmogenic cardiomyopathy patients. J Vis Exp.

[33] Huang GTJ, Gronthos S, Shi S (2009). Mesenchymal stem cells derived from dental tissues vs. those from other sources: their biology and role in regenerative medicine. J Dent Res.

[34] Ledesma-Martínez E, Mendoza-Núñez VM, Santiago-Osorio E (2016). Mesenchymal stem cells derived from dental pulp: a review. Stem Cells Int.

[35] Čamernik K, Mihelič A, Mihalič R, Marolt Presen D, Janež A, Trebše R, Marc J, Zupan J (2019). Skeletal-muscle-derived mesenchymal stem/stromal cells from patients with osteoarthritis show superior biological properties compared to bone-derived cells. Stem Cell Res.

[36] M Ahmed S, El-Badri N. Pancreatic mesenchymal stromal cells: characteristics and possible origin. Liver Pancreatic Sci. 2016;1(1).

[37] Martinu T, Palmer SM, Ortiz LA (2011). Lung-resident mesenchymal stromal cells. Am J Respir Crit Care Med.

[38] Hawkins KE, Corcelli M, Dowding K, Ranzoni AM, Vlahova F, Hau KL, Hunjan A, Peebles D, Gressens P, Hagberg H (2018). Embryonic stem cell-derived mesenchymal stem cells (MSCs) have a superior neuroprotective capacity over fetal MSCs in the hypoxic-ischemic mouse brain. Stem Cells Transl Med.

[39] Xu M, Shaw G, Murphy M, Barry F (2019). Induced pluripotent stem cell-derived mesenchymal stromal cells are functionally and genetically different from bone marrow-derived mesenchymal stromal cells. Stem Cells.

[40] Pittenger MF, Mackay AM, Beck SC, Jaiswal RK, Douglas R, Mosca JD, Moorman MA, Simonetti DW, Craig S, Marshak DR (1999). Multilineage potential of adult human mesenchymal stem cells. Science.

[41] Matikainen T, Laine J (2005). Placenta--an alternative source of stem cells. Toxicol Appl Pharmacol.

[42] de la Torre P, Jesús Pérez-Lorenzo MI, Flores A (2019). Human placenta-derived mesenchymal stromal cells: a review from basic research to clinical applications.

[43] Iwatani S, Yoshida M, Yamana K, Kurokawa D, Kuroda J, Thwin KKM, Uemura S, Takafuji S, Nino N, Koda T, et al. Isolation and characterization of human umbilical cord-derived mesenchymal stem cells from preterm and term infants. J Vis Exp. 2019;143. 10.3791/58806 30741254

[44] Divya MS, Roshin GE, Divya TS, Rasheed VA, Santhoshkumar TR, Elizabeth KE, James J, Pillai RM (2012). Umbilical cord blood-derived mesenchymal stem cells consist of a unique population of progenitors co-expressing mesenchymal stem cell and neuronal markers capable of instantaneous neuronal differentiation. Stem Cell Res Ther.

[45] González PL, Carvajal C, Cuenca J, Alcayaga-Miranda F, Figueroa FE, Bartolucci J, Salazar-Aravena L, Khoury M (2015). Chorion mesenchymal stem cells show superior differentiation, immunosuppressive, and angiogenic potentials in comparison with haploidentical maternal placental cells. Stem Cells Transl Med.

[46] Jiao F, Wang J, Dong Z-L, Wu M-J, Zhao T-B, Li D-D, Wang X (2012). Human mesenchymal stem cells derived from limb bud can differentiate into all three embryonic germ layers lineages. Cell Reprogram.

[47] Marin-Llera JC, Chimal-Monroy J (2018). A small population of resident limb bud mesenchymal cells express few MSC-associated markers, but the expression of these markers is increased immediately after cell culture. Cell Biol Int.

[48] Cheng Y, Li L, Wang D, Guo Q, He Y, Liang T, Sun L, Wang X, Cheng Y, Zhang G (2017). Characteristics of human endometrium-derived mesenchymal stem cells and their tropism to endometriosis. Stem Cells Int.

[49] Mutlu L, Hufnagel D, Taylor HS (2015). The endometrium as a source of mesenchymal stem cells for regenerative medicine. Biol Reprod.

[50] Corradetti B, Correani A, Romaldini A, Marini MG, Bizzaro D, Perrini C, Cremonesi F, Lange-Consiglio A (2014). Amniotic membrane-derived mesenchymal cells and their conditioned media: potential candidates for uterine regenerative therapy in the horse. PLoS One.

[51] Thilakavathy K, Nordin N, Ramasamy R, Ghoraishizadeh P, IMR R, Singh G (2017). Characteristics of full-term amniotic fluid-derived mesenchymal stem cells in different culture media.

[52] Dominguez-Bendala J, Lanzoni G, Inverardi L, Ricordi C (2012). Concise review: mesenchymal stem cells for diabetes. Stem Cells Transl Med.

[53] Bhansali A, Upreti V, Khandelwal N, Marwaha N, Gupta V, Sachdeva N, Sharma RR, Saluja K, Dutta P, Walia R (2009). Efficacy of autologous bone marrow-derived stem cell transplantation in patients with type 2 diabetes mellitus. Stem Cells Dev.

[54] Bhansali A, Asokumar P, Walia R, Bhansali S, Gupta V, Jain A, Sachdeva N, Sharma RR, Marwaha N, Khandelwal N (2014). Efficacy and safety of autologous bone marrow-derived stem cell transplantation in patients with type 2 diabetes mellitus: a randomized placebo-controlled study. Cell Transpl.

[55] Wei H, Tan G, Manasi QS, Kong G, Yong P, Koh C, Ooi TH, Lim SY, Wong P (2012). One-step derivation of cardiomyocytes and mesenchymal stem cells from human pluripotent stem cells. Stem Cell Res.

[56] Lian Q, Zhang Y, Zhang J, Zhang Hua K, Wu X, Zhang Y, Lam Francis F-Y, Kang S, Xia Jian C, Lai W-H (2010). Functional mesenchymal stem cells derived from human induced pluripotent stem cells attenuate limb ischemia in mice. Circulation.

[57] Salama E, Eldeen GN, Abdel Rasheed M, Abdel Atti S, Elnoury A, Taha T, Azmy O (2018). Differentially expressed genes: OCT-4, SOX2, STAT3, CDH1 and CDH2, in cultured mesenchymal stem cells challenged with serum of women with endometriosis. J Genet Eng Biotechnol.

[58] Cho J, D'Antuono M, Glicksman M, Wang J, Jonklaas J (2018). A review of clinical trials: mesenchymal stem cell transplant therapy in type 1 and type 2 diabetes mellitus. Am J Stem Cells.

[59] Mu X-P, Ren L-Q, Yan H-W, Zhang X-M, Xu T-M, Wei A-H, Jiang J-L (2017). Enhanced differentiation of human amniotic fluid-derived stem cells into insulin-producing cells in vitro. J Diabetes Investig.

[60] S Parekh V, Joglekar M, Hardikar A (2009). Differentiation of human umbilical cord blood-derived mononuclear cells to endocrine pancreatic lineage, vol. 78.

[61] Kadam S, Muthyala S, Nair P, Bhonde R (2010). Human placenta-derived mesenchymal stem cells and islet-like cell clusters generated from these cells as a novel source for stem cell therapy in diabetes. Rev Diabet Stud.

[62] Chatterjee T, Sarkar RS, Dhot PS, Kumar S, Kumar VK (2010). Adult stem cell plasticity: dream or reality?. Med J Armed Forces India.

[63] Ma J, Wu J, Han L, Jiang X, Yan L, Hao J, Wang H (2019). Comparative analysis of mesenchymal stem cells derived from amniotic membrane, umbilical cord, and chorionic plate under serum-free condition. Stem Cell Res Ther.

[64] Gao F, Wu DQ, Hu YH, Jin GX (2008). Extracellular matrix gel is necessary for in vitro cultivation of insulin producing cells from human umbilical cord blood derived mesenchymal stem cells. Chin Med J.

[65] Sun B, Roh KH, Lee SR, Lee YS, Kang KS (2007). Induction of human umbilical cord blood-derived stem cells with embryonic stem cell phenotypes into insulin producing islet-like structure. Biochem Biophys Res Commun.

[66] Gao F, Wu D-Q, Hu Y-H, Jin G-X, Li G-D, Sun T-W, Li F-J (2008). In vitro cultivation of islet-like cell clusters from human umbilical cord blood-derived mesenchymal stem cells. Transl Res.

[67] Santos TM, Percegona LS, Gonzalez P, Calil A, Corradi Perini C, Faucz FR, Camara NO, Aita CA (2010). Expression of pancreatic endocrine markers by mesenchymal stem cells from human umbilical cord vein. Transpl Proc.

[68] Nguyen P, Nguyen A, Nguyen N, Nguyen N, Duong T, Truong N, Phan N (2014). Human umbilical cord blood derived mesenchymal stem cells were differentiated into pancreatic endocrine cell by Pdx-1 electrotransfer. Biomed Res Ther.

[69] Nekoei SM, Azarpira N, Sadeghi L, Kamalifar S (2015). In vitro differentiation of human umbilical cord Wharton’s jelly mesenchymal stromal cells to insulin producing clusters. World J Clin Cases.

[70] Xin Y, Jiang X, Wang Y, Su X, Sun M, Zhang L, Tan Y, Wintergerst KA, Li Y, Li Y (2016). Insulin-producing cells differentiated from human bone marrow mesenchymal stem cells in vitro ameliorate streptozotocin-induced diabetic hyperglycemia. PLoS One.

[71] Xie QP, Huang H, Xu B, Dong X, Gao SL, Zhang B, Wu YL (2009). Human bone marrow mesenchymal stem cells differentiate into insulin-producing cells upon microenvironmental manipulation in vitro. Differentiation.

[72] Karnieli O, Izhar-Prato Y, Bulvik S, Efrat S (2007). Generation of insulin-producing cells from human bone marrow mesenchymal stem cells by genetic manipulation. Stem Cells.

[73] Tang DQ, Wang Q, Burkhardt BR, Litherland SA, Atkinson MA, Yang LJ (2012). In vitro generation of functional insulin-producing cells from human bone marrow-derived stem cells, but long-term culture running risk of malignant transformation. Am J Stem Cells.

[74] Gabr MM, Zakaria MM, Refaie AF, Ismail AM, Abou-El-Mahasen MA, Ashamallah SA, Khater SM, El-Halawani SM, Ibrahim RY, Uin GS (2013). Insulin-producing cells from adult human bone marrow mesenchymal stem cells control streptozotocin-induced diabetes in nude mice. Cell Transpl.

[75] Jafarian A, Taghikani M, Abroun S, Allahverdi A, Lamei M, Lakpour N, Soleimani M (2015). The generation of insulin producing cells from human mesenchymal stem cells by MiR-375 and anti-MiR-9. PLoS One.

[76] Han Y, Kim J, Yang J, Kim W, Zhou X (2016). Method of inducing beta cells from urine-derived cells using small molecules. Google patents.

[77] Shivakumar SB, Lee H-J, Son Y-B, Bharti D, Ock SA, Lee S-L, Kang Y-H, Park B-W, Rho G-J (2019). In vitro differentiation of single donor derived human dental mesenchymal stem cells into pancreatic β cell-like cells. Biosci Rep.

[78] Chandra V, Swetha G, Muthyala S, Jaiswal AK, Bellare JR, Nair PD, Bhonde RR (2011). Islet-like cell aggregates generated from human adipose tissue derived stem cells ameliorate experimental diabetes in mice. PLoS One.

[79] Li Y, Zhang R, Qiao H, Zhang H, Wang Y, Yuan H, Liu Q, Liu D, Chen L, Pei X (2007). Generation of insulin-producing cells from PDX-1 gene-modified human mesenchymal stem cells. J Cell Physiol.

[80] Phadnis SM, Joglekar MV, Dalvi MP, Muthyala S, Nair PD, Ghaskadbi SM, Bhonde RR, Hardikar AA (2011). Human bone marrow-derived mesenchymal cells differentiate and mature into endocrine pancreatic lineage in vivo. Cytotherapy.

[81] Gabr MM, Zakaria MM, Refaie AF, Khater SM, Ashamallah SA, Ismail AM, El-Badri N, Ghoneim MA (2014). Generation of insulin-producing cells from human bone marrow-derived mesenchymal stem cells: comparison of three differentiation protocols. Biomed Res Int.

[82] Moriscot C, de Fraipont F, Richard MJ, Marchand M, Savatier P, Bosco D, Favrot M, Benhamou PY (2005). Human bone marrow mesenchymal stem cells can express insulin and key transcription factors of the endocrine pancreas developmental pathway upon genetic and/or microenvironmental manipulation in vitro. Stem Cells.

[83] Timper K, Seboek D, Eberhardt M, Linscheid P, Christ-Crain M, Keller U, Müller B, Zulewski H (2006). Human adipose tissue-derived mesenchymal stem cells differentiate into insulin, somatostatin, and glucagon expressing cells. Biochem Biophys Res Commun.

[84] Jung Da-Woon, Kim Woong-Hee, Williams Darren Reece (2013). Reprogram or Reboot: Small Molecule Approaches for the Production of Induced Pluripotent Stem Cells and Direct Cell Reprogramming. ACS Chemical Biology.

[85] Oh SH, Muzzonigro TM, Bae SH, LaPlante JM, Hatch HM, Petersen BE (2004). Adult bone marrow-derived cells trans-differentiating into insulin-producing cells for the treatment of type I diabetes. Lab Investig.

[86] Efe JA, Ding S (2011). The evolving biology of small molecules: controlling cell fate and identity. Philos Trans R Soc London B Biol Sci.

[87] Lin T, Wu S (2015). Reprogramming with small molecules instead of exogenous transcription factors. Stem Cells Int.

[88] Liu D, Pavathuparambil Abdul Manaph N, Al-Hawwas M, Zhou XF, Liao H (2018). Small molecules for neural stem cell induction. Stem Cells Dev.

[89] Olivier EN, Marenah L, McCahill A, Condie A, Cowan S, Mountford JC (2016). High-efficiency serum-free feeder-free erythroid differentiation of human pluripotent stem cells using small molecules. Stem Cells Transl Med.

[90] Ao A, Hao J, Hong CC (2011). Regenerative chemical biology: current challenges and future potential. Chem Biol.

[91] Kumar SS, Alarfaj AA, Munusamy MA, Singh AJ, Peng IC, Priya SP, Hamat RA, Higuchi A (2014). Recent developments in beta-cell differentiation of pluripotent stem cells induced by small and large molecules. Int J Mol Sci.

[92] Borowiak M (2010). The new generation of beta-cells: replication, stem cell differentiation, and the role of small molecules. Revi Diabetic Stud.

[93] Al Madhoun A, Ali H, AlKandari S, Atizado VL, Akhter N, Al-Mulla F, Atari M (2016). Defined three-dimensional culture conditions mediate efficient induction of definitive endoderm lineage from human umbilical cord Wharton’s jelly mesenchymal stem cells. Stem Cell Res Ther.

[94] Kroon E, Martinson LA, Kadoya K, Bang AG, Kelly OG, Eliazer S, Young H, Richardson M, Smart NG, Cunningham J (2008). Pancreatic endoderm derived from human embryonic stem cells generates glucose-responsive insulin-secreting cells in vivo. Nat Biotech.

[95] Rezania A, Bruin JE, Riedel MJ, Mojibian M, Asadi A, Xu J, Gauvin R, Narayan K, Karanu F, O'Neil JJ (2012). Maturation of human embryonic stem cell-derived pancreatic progenitors into functional islets capable of treating pre-existing diabetes in mice. Diabetes.

[96] Xie R, Everett LJ, Lim HW, Patel NA, Schug J, Kroon E, Kelly OG, Wang A, D'Amour KA, Robins AJ (2013). Dynamic chromatin remodeling mediated by polycomb proteins orchestrates pancreatic differentiation of human embryonic stem cells. Cell Stem Cell.

[97] Narayanan K, Lim VY, Shen J, Tan ZW, Rajendran D, Luo SC, Gao S, Wan AC, Ying JY (2014). Extracellular matrix-mediated differentiation of human embryonic stem cells: differentiation to insulin-secreting beta cells. Tissue Eng Part A.

[98] Kerby A, Jones ES, Jones PM, King AJ (2013). Co-transplantation of islets with mesenchymal stem cells in microcapsules demonstrates graft outcome can be improved in an isolated-graft model of islet transplantation in mice. Cytotherapy.

[99] Vaithilingam V, Evans MDM, Lewy DM, Bean PA, Bal S, Tuch BE (2017). Co-encapsulation and co-transplantation of mesenchymal stem cells reduces pericapsular fibrosis and improves encapsulated islet survival and function when allografted. Sci Rep.

[100] Shibata T, Naruse K, Kamiya H, Kozakae M, Kondo M, Yasuda Y, Nakamura N, Ota K, Tosaki T, Matsuki T (2008). Transplantation of bone marrow–derived mesenchymal stem cells improves diabetic polyneuropathy in rats. Diabetes.

[101] Hu J, Wang Y, Gong H, Yu C, Guo C, Wang F, Yan S, Xu H (2016). Long term effect and safety of Wharton’s jelly-derived mesenchymal stem cells on type 2 diabetes. Exp therapeutic Med.

[102] Pagliuca FW, Melton DA (2013). How to make a functional beta-cell. Development.

[103] Abdelalim EM, Emara MM (2015). Advances and challenges in the differentiation of pluripotent stem cells into pancreatic beta cells. World J Stem Cells.

[104] Taylor BL, Liu F-F, Sander M (2013). Nkx6.1 is essential for maintaining the functional state of pancreatic beta cells. Cell Rep.

[105] Russ HA, Parent AV, Ringler JJ, Hennings TG, Nair GG, Shveygert M, Guo T, Puri S, Haataja L, Cirulli V (2015). Controlled induction of human pancreatic progenitors produces functional beta-like cells in vitro. EMBO J.

[106] Liu XD, Ruan JX, Xia JH, Yang SL, Fan JH, Li K (2014). The study of regulatory effects of Pdx-1, MafA and NeuroD1 on the activity of porcine insulin promoter and the expression of human islet amyloid polypeptide. Mol Cell Biochem.

[107] Lee CS, Sund NJ, Vatamaniuk MZ, Matschinsky FM, Stoffers DA, Kaestner KH (2002). Foxa2 controls Pdx1 gene expression in pancreatic beta-cells in vivo. Diabetes.

[108] Bastidas-Ponce A, Roscioni SS, Burtscher I, Bader E, Sterr M, Bakhti M, Lickert H (2017). Foxa2 and Pdx1 cooperatively regulate postnatal maturation of pancreatic β-cells. Mol Metab.

[109] Yang SF, Xue WJ, Duan YF, Xie LY, Lu WH, Zheng J, Yin AP (2015). Nicotinamide facilitates mesenchymal stem cell differentiation into insulin-producing cells and homing to pancreas in diabetic mice. Transplant Proc.

[110] Dave S (2014). Mesenchymal stem cells derived in vitro transdifferentiated insulin-producing cells: a new approach to treat type 1 diabetes. Adv Biomed Res.

[111] Cho YM, Lim JM, Yoo DH, Kim JH, Chung SS, Park SG, Kim TH, Oh SK, Choi YM, Moon SY (2008). Betacellulin and nicotinamide sustain PDX1 expression and induce pancreatic beta-cell differentiation in human embryonic stem cells. Biochem Biophys Res Commun.

[112] Paz AH, Salton GD, Ayala-Lugo A, Gomes C, Terraciano P, Scalco R, Laurino CC, Passos EP, Schneider MR, Meurer L (2011). Betacellulin overexpression in mesenchymal stem cells induces insulin secretion in vitro and ameliorates streptozotocin-induced hyperglycemia in rats. Stem Cells Dev.

[113] Sirchia SM, Faversani A, Rovina D, Russo MV, Paganini L, Savi F, Augello C, Rosso L, Gobbo AD, Tabano S (2016). Epigenetic effects of chromatin remodeling agents on organotypic cultures. Epigenomics.

[114] Thatava T, Ma B, Rohde M, Mayer H (2006). Chromatin-remodeling factors allow differentiation of bone marrow cells into insulin-producing cells. Stem Cells.

[115] Jopling C, Boue S, Izpisua Belmonte JC (2011). Dedifferentiation, transdifferentiation and reprogramming: three routes to regeneration. Nat Rev Mol Cell Biol.

[116] Borowiak M, Maehr R, Chen S, Chen AE, Tang W, Fox JL, Schreiber SL, Melton DA (2009). Small molecules efficiently direct endodermal differentiation of mouse and human embryonic stem cells. Cell Stem Cell.

[117] Carpenter MK, Rao MS (2015). Concise review: making and using clinically compliant pluripotent stem cell lines. Stem Cells Transl Med.

[118] Al-Khawaga S, Memon B, Butler AE, Taheri S, Abou-Samra AB, Abdelalim EM (2018). Pathways governing development of stem cell-derived pancreatic beta cells: lessons from embryogenesis. Biol Rev Camb Philos Soc.

[119] Deering TG, Ogihara T, Trace AP, Maier B, Mirmira RG (2009). Methyltransferase Set7/9 maintains transcription and euchromatin structure at islet-enriched genes. Diabetes.

[120] Baharvand H, Jafary H, Massumi M, Ashtiani SK (2006). Generation of insulin-secreting cells from human embryonic stem cells. Develop Growth Differ.

[121] Pagliuca Felicia W, Millman Jeffrey R, Gürtler M, Segel M, Van Dervort A, Ryu Jennifer H, Peterson Quinn P, Greiner D, Melton DA (2014). Generation of functional human pancreatic β cells in vitro. Cell.

[122] Galipeau J, Sensebe L (2018). Mesenchymal stromal cells: clinical challenges and therapeutic opportunities. Cell Stem Cell.

[123] Wu LF, Wang NN, Liu YS, Wei X (2009). Differentiation of Wharton’s jelly primitive stromal cells into insulin-producing cells in comparison with bone marrow mesenchymal stem cells. Tissue Eng Part A.

[124] Johannesson B, Sui L, Freytes DO, Creusot RJ, Egli D (2015). Toward beta cell replacement for diabetes. EMBO J.

[125] Shapiro AM, Ricordi C, Hering BJ, Auchincloss H, Lindblad R, Robertson RP, Secchi A, Brendel MD, Berney T, Brennan DC (2006). International trial of the Edmonton protocol for islet transplantation. N Engl J Med.

[126] Mayhew CN, Wells JM (2010). Converting human pluripotent stem cells into beta-cells: recent advances and future challenges. Curr Opin Organ Transpl.

[127] Ma X, Zhu S (2017). Chemical strategies for pancreatic β cell differentiation, reprogramming, and regeneration. Acta Biochim Biophys Sin.

[128] Hoveizi E, Tavakol S (2019). Therapeutic potential of human mesenchymal stem cells derived beta cell precursors on a nanofibrous scaffold: an approach to treat diabetes mellitus. J Cell Physiol.

[129] Gourgari E, Wilhelm EE, Hassanzadeh H, Aroda VR, Shoulson I (2017). A comprehensive review of the FDA-approved labels of diabetes drugs: indications, safety, and emerging cardiovascular safety data. J Diabetes Complicat.

[130] Ullah I, Subbarao RB, Rho GJ (2015). Human mesenchymal stem cells - current trends and future prospective. Biosci Rep.

[131] Prasajak P, Leeanansaksiri W (2013). Developing a new two-step protocol to generate functional hepatocytes from Wharton’s jelly-derived mesenchymal stem cells under hypoxic condition. Stem Cells Int.

[132] Schmöle A-C, Hübner R, Beller M, Rolfs A, Frech MJ (2013). Small molecules in stem cell research.

[133] Ripps H, Shen W (2012). Review: taurine: a “very essential” amino acid. Mol Vis.

[134] Esteban MA, Wang T, Qin B, Yang J, Qin D, Cai J, Li W, Weng Z, Chen J, Ni S (2010). Vitamin C enhances the generation of mouse and human induced pluripotent stem cells. Cell Stem Cell.

[135] Chen S, Borowiak M, Fox JL, Maehr R, Osafune K, Davidow L, Lam K, Peng LF, Schreiber SL, Rubin LL (2009). A small molecule that directs differentiation of human ESCs into the pancreatic lineage. Nat Chem Biol.

[136] Maehr R, Chen S, Snitow M, Ludwig T, Yagasaki L, Goland R, Leibel RL, Melton DA (2009). Generation of pluripotent stem cells from patients with type 1 diabetes. Proc Natl Acad Sci.

[137] Rezania A, Bruin JE, Arora P, Rubin A, Batushansky I, Asadi A, O'Dwyer S, Quiskamp N, Mojibian M, Albrecht T (2014). Reversal of diabetes with insulin-producing cells derived in vitro from human pluripotent stem cells. Nat Biotechnol.

[138] Birkenkamp KU, Tuyt LML, Lummen C, Wierenga ATJ, Kruijer W, Vellenga E (2000). The p38 MAP kinase inhibitor SB203580 enhances nuclear factor-kappa B transcriptional activity by a non-specific effect upon the ERK pathway. Br J Pharmacol.

[139] Snykers S, Vanhaecke T, De Becker A, Papeleu P, Vinken M, Van Riet I, Rogiers V (2007). Chromatin remodeling agent trichostatin A: a key-factor in the hepatic differentiation of human mesenchymal stem cells derived of adult bone marrow. BMC Dev Biol.

[140] Zhou M, Li P, Tan L, Qu S, Ying QL, Song H (2010). Differentiation of mouse embryonic stem cells into hepatocytes induced by a combination of cytokines and sodium butyrate. J Cell Biochem.

[141] Perez DI, Palomo V, Perez C, Gil C, Dans PD, Luque FJ, Conde S, Martinez A (2011). Switching reversibility to irreversibility in glycogen synthase kinase 3 inhibitors: clues for specific design of new compounds. J Med Chem.

[142] Barrett SD, Bridges AJ, Dudley DT, Saltiel AR, Fergus JH, Flamme CM, Delaney AM, Kaufman M, LePage S, Leopold WR (2008). The discovery of the benzhydroxamate MEK inhibitors CI-1040 and PD 0325901. Bioorg Med Chem Lett.

[143] Sanvitale CE, Kerr G, Chaikuad A, Ramel MC, Mohedas AH, Reichert S, Wang Y, Triffitt JT, Cuny GD, Yu PB (2013). A new class of small molecule inhibitor of BMP signaling. PLoS One.

[144] Frankland PW, Josselyn SA, Bradwejn J, Vaccarino FJ, Yeomans JS (1996). Intracerebroventricular infusion of the CCKB receptor agonist pentagastrin potentiates acoustic startle. Brain Res.

[145] Knudsen LB, Kiel D, Teng M, Behrens C, Bhumralkar D, Kodra JT, Holst JJ, Jeppesen CB, Johnson MD, de Jong JC (2007). Small-molecule agonists for the glucagon-like peptide 1 receptor. Proc Natl Acad Sci.

[146] Manandhar B, Ahn J-M (2015). Glucagon-like peptide-1 (GLP-1) analogs: recent advances, new possibilities, and therapeutic implications. J Med Chem.

[147] Gupta V (2013). Glucagon-like peptide-1 analogues: an overview. Indian J Endocrinol Metab.

